# Incomplete Conflict of Interest Disclosures

**DOI:** 10.1001/jamanetworkopen.2024.26687

**Published:** 2024-06-24

In 4 articles published in *JAMA Network Open* between 2018 and 2021,^1,2,3,4^ conflict of interest disclosures for David B. Abrams, PhD, and Raymond S. Niaura, PhD, were omitted or incomplete. These authors have provided additional disclosures, which include the following. For Dr Abrams, “other NIDA grants paid to his employers; receiving salary from the Steven Schroeder Institute for Tobacco Research and Policy Studies at The Legacy Foundation, now Truth Initiative, New York University School of Global Public Health; and between mid-2015 and 2020, frequently communicating with Juul Labs personnel, for which there was no compensation, and receiving hospitality in the form of meals at some meetings.” For Dr Niaura, “serving as a paid consultant to the Government of Canada via a contract with Industrial Economics Inc; receiving an honorarium for a virtual meeting from Pfizer Inc; receiving other NIDA grants paid to his employers; receiving salary from the Steven Schroeder Institute for Tobacco Research and Policy Studies at The Legacy Foundation, now Truth Initiative, New York University School of Global Public Health; and between mid-2015 and 2020, frequently communicating with Juul Labs personnel, for which there was no compensation, and receiving hospitality in the form of meals at some meetings.” These articles have been corrected to include these additional disclosures.^1,2,3,4^
